# Mindfulness-based therapy for chronic tinnitus: a narrative review

**DOI:** 10.3389/fpsyg.2026.1738053

**Published:** 2026-05-08

**Authors:** Zhongfu Zhang, Qingqin Xu, Jing Wang, Yongxin Luo, Qianer Shao, Yang Jinhua Li, Haoyu Lu, Jianwei Lu

**Affiliations:** Laboratory of Traditional Chinese Medicine, Shanghai University of Traditional Chinese Medicine, Shanghai, China

**Keywords:** chronic tinnitus, mindfulness practice, mindfulness therapy, narrative review, psychological intervention

## Abstract

Chronic tinnitus is often accompanied by considerable psychological burden, including anxiety, depression, and impaired quality of life. Mindfulness-based therapy has attracted growing attention as a potential psychological intervention for tinnitus management. This narrative review examines the current literature on the rationale, clinical application, reported efficacy, limitations, and future directions of mindfulness-based therapy in chronic tinnitus. Current evidence suggests that mindfulness-based interventions may help reduce tinnitus-related distress, improve emotional regulation, and enhance quality of life, while evidence for direct effects on tinnitus loudness remains limited. Although findings from existing studies are encouraging, the literature is heterogeneous with respect to intervention types, comparator conditions, and outcome measures. Mindfulness-based therapy may therefore represent a useful adjunctive option for some patients with chronic tinnitus, but further rigorous research is required to better define its clinical role.

## 1 Introduction

Tinnitus is defined as the perception of sound in the absence of an external acoustic stimulus and is commonly described as ringing, buzzing, or hissing in the ears or head. It is generally considered chronic when it persists for more than 3 months. Although tinnitus is a symptom rather than a disease entity in itself, its clinical significance should not be underestimated. For many individuals, chronic tinnitus is associated with substantial tinnitus-related distress, impaired quality of life, sleep disturbance, difficulties with concentration, and emotional symptoms such as anxiety and depression, all of which may interfere with daily functioning (Tinnitus, 2021). Epidemiological studies indicate that approximately 25% of adults in Western countries have experienced tinnitus episodes, with 10%−15% of individuals suffering from chronic tinnitus over the long term (Henry et al., 2020). Current management approaches for chronic tinnitus include treatment of identifiable underlying causes, pharmacological approaches, rehabilitative strategies, and psychological interventions. When an underlying cause can be identified, targeted treatment may help reduce tinnitus burden in some cases. However, the etiology of tinnitus is often heterogeneous and multifactorial, and in many patients a clear reversible cause cannot be established, which limits the effectiveness of purely aetiological treatment (Shore et al., 2016). Pharmacological approaches have also been investigated, but no medication has been shown to consistently eliminate chronic tinnitus, and reported benefits are generally limited or inconsistent (Kim et al., 2021; Langguth and Elgoyhen, 2012). Rehabilitative approaches, such as sound-based interventions, photobiomodulation, and electrical stimulation, have also been explored. Although some of these approaches may help reduce tinnitus-related burden in selected patients, their long-term effectiveness, standardization, and consistency across patient groups remain uncertain (Choi et al., 2024; Lee et al., 2024; Martins et al., 2022; Talluri et al., 2022; Thabit et al., 2014). Taken together, these limitations highlight an important clinical challenge: while some existing approaches may provide partial symptomatic relief or support adaptation, effective treatments that directly reduce the tinnitus percept itself remain limited. As a result, increasing attention has been directed toward psychological interventions that aim not necessarily to eliminate the sound, but to reduce tinnitus-related distress and improve patients' ability to cope with the condition.

Psychological therapy is an integral component of chronic tinnitus treatment, aimed at alleviating tinnitus symptoms and enhancing patients' psychological adaptability. Common psychological therapies include cognitive behavioral therapy (CBT), relaxation training (RT), tinnitus retraining therapy (TRT), and mindfulness-based interventions (MBI; Mekki et al., 2024). Among these, mindfulness-based interventions have received growing attention as a potential approach for addressing the emotional and cognitive burden associated with chronic tinnitus. Rather than altering tinnitus loudness directly, mindfulness-based approaches are generally understood to help patients change how they attend to, interpret, and emotionally respond to the tinnitus percept. In this way, they may reduce tinnitus-related distress, support emotional regulation, and improve quality of life. Although some studies have suggested that mindfulness-based interventions may be beneficial for patients with chronic tinnitus (Ost, 2008), the current evidence should be interpreted with caution. Reported outcomes vary substantially across studies, and the observed benefits appear to relate primarily to tinnitus-related distress and associated psychological functioning rather than to direct reductions in tinnitus loudness. In addition, although neurocognitive and neuroimaging correlates of mindfulness-based interventions have been explored, these findings remain preliminary and should not be taken as definitive evidence of a specific mechanism of action (Creswell, 2017). Accordingly, the potential value of mindfulness-based interventions in chronic tinnitus is most appropriately understood in terms of their effects on attention, emotional regulation, acceptance, and coping.

Although several reviews and meta-analyses have examined psychological treatments for tinnitus, many have focused primarily on cognitive behavioural therapy or on broader psychological intervention frameworks. In addition, recent developments, including online or digital delivery of mindfulness-based interventions and emerging neuroimaging findings, have not been systematically synthesised in an up-to-date and critical manner. The present review therefore aims to provide a critical synthesis of mindfulness-based interventions for chronic tinnitus. Rather than arguing that mindfulness directly reduces tinnitus loudness, this review examines the extent to which these interventions may alleviate tinnitus-related distress and broader psychological burden, clarifies the conceptual boundaries of the existing evidence, and identifies important limitations and future research priorities. This focus is timely given continuing clinical interest in non-pharmacological approaches to chronic tinnitus, the expansion of digitally and remotely delivered mindfulness programmes, and the growing, yet still methodologically limited, body of research in this area.

This article was conducted as a narrative review of the current literature on mindfulness-based interventions for chronic tinnitus. Relevant studies were identified through a structured search of PubMed, Web of Science, and Scopus using terms related to “chronic tinnitus”, “mindfulness”, “mindfulness-based stress reduction”, and “mindfulness-based cognitive therapy”. Priority was given to English-language studies addressing the conceptual rationale, clinical application, reported outcomes, methodological limitations, and future directions of mindfulness-based interventions in chronic tinnitus. Where relevant, selected studies from related areas were also considered to contextualize proposed mechanisms or psychological effects. As a narrative review, this article was not designed to follow a formal PRISMA-based systematic review process or to provide a quantitative pooled estimate of treatment effect. Instead, its purpose is to offer a critical synthesis of the available literature, with particular attention to conceptual precision, differences in outcome constructs, variation in study quality, and the distinction between established findings and more preliminary or speculative lines of inquiry. Studies were considered relevant if they examined mindfulness-based interventions in chronic tinnitus or provided clinically meaningful background on mindfulness-related psychological mechanisms that could plausibly inform tinnitus care. Studies focusing exclusively on non-tinnitus conditions without clear relevance to the aims of this review were not prioritized.

## 2 Chronic tinnitus

### 2.1 Chronic tinnitus overview

Tinnitus is commonly defined as the perception of sound in the absence of an external acoustic stimulus (Marion and Cevette, 1991). It may be experienced as ringing, buzzing, hissing, or other phantom sounds, and can occur either continuously or intermittently, with perceived intensity and character varying across individuals and contexts (Baguley et al., 2013). Clinically, tinnitus is often classified as either objective or subjective. Objective tinnitus is relatively uncommon and arises from internal sound sources that may, in some cases, be detectable by an examiner and amenable to targeted treatment. By contrast, subjective tinnitus, particularly chronic subjective tinnitus, refers to the perception of sound without an identifiable external source and is the form most commonly encountered in clinical practice (Ryan and Bauer, 2016). Chronic tinnitus is generally considered to persist for more than 3 months and represents a substantial clinical and public health concern. Epidemiological studies suggest that approximately 10%−15% of the population experience chronic tinnitus, with prevalence tending to increase with age (Biswas et al., 2022). Although tinnitus is highly prevalent, its clinical impact is not determined solely by the auditory percept itself. A substantial proportion of patients report significant disruption to daily life, including difficulties with sleep, concentration, emotional well being, and overall quality of life (Dalrymple et al., 2021), Importantly, the burden of tinnitus is often related more to tinnitus-related distress and emotional responses than to the perceived loudness of the sound itself. The etiology of tinnitus is complex and may involve ear-related disorders, neurological dysfunction, or systemic diseases. Although treating underlying conditions can alleviate tinnitus in some cases, such approaches are often limited by the multifactorial nature of tinnitus and substantial inter-individual variability in treatment response. Additionally, chronic tinnitus often occurs secondary to other conditions, such as temporomandibular joint disorders, cervical spine injuries, and is frequently associated with sleep disorders, depression, or anxiety (Baguley et al., 2013).

### 2.2 Main treatment methods for chronic tinnitus

Patients with chronic tinnitus often experience difficulties in emotional regulation, maladaptive behavioral responses, and impairments in daily functioning and communication (Mazurek et al., 2022). Although multiple treatment options are available, no single intervention has consistently demonstrated sustained benefit across all patient groups (Czornik et al., 2022; Langguth, 2015). Current management strategies for chronic tinnitus generally include etiological treatment, pharmacological therapy, rehabilitation approaches, and psychological interventions.

Etiological treatment aims to address underlying medical conditions that may contribute to tinnitus (Van de Heyning et al., 2015). The causes of tinnitus are heterogeneous and may involve otological, neurological, and systemic conditions. In some cases, treating underlying ear-related disorders may help reduce tinnitus perception (Li et al., 2022). Neurological dysfunction may also contribute to tinnitus in certain patients. In such cases, treatment typically focuses on managing the underlying neurological abnormality rather than directly targeting tinnitus itself (Cil et al., 2020; Schreiber et al., 2010). In addition, Systemic diseases may indirectly contribute to tinnitus through effects on circulation or neural function. For these cases, controlling the progression of the underlying condition and treating the primary disease are key to improving tinnitus symptoms (Shapiro et al., 2021; Sismanis, 2011). However, even when a potential cause is identified, treatment outcomes are often limited by the multifactorial nature of tinnitus and marked inter-individual variability in response. Moreover, some etiological interventions may have limited durability or may not be suitable for all patients (Baguley et al., 2013). Overall, etiological treatment may be beneficial in selected patients, but its clinical utility is often constrained by etiological complexity and variability in treatment response (Narsinh et al., 2022).

Pharmacological treatment also has a limited role in chronic tinnitus management. At present, no medication has been specifically approved for chronic tinnitus (Langguth and Elgoyhen, 2011). In practice, pharmacological agents are more commonly used to manage associated symptoms, such as anxiety, depression, or sleep disturbance, rather than to directly reduce the tinnitus percept itself (Fornaro and Martino, 2010; Hoekstra et al., 2011; Kim et al., 2021; Sereda et al., 2023; Vijayakumar et al., 2022). Their effects are variable, and potential adverse effects, together with the lack of robust long-term evidence from large randomized trials, further constrain their clinical value (Bauer, 2020; Folmer et al., 2020; Mazurek et al., 2022; Palumbo et al., 2015; Sismanis, 2001).

Common rehabilitative approaches for chronic tinnitus include sound therapy, photobiomodulation, and electrical stimulation. Sound therapy is generally intended to reduce the prominence of tinnitus by facilitating habituation or by providing external auditory input that may lessen attention to tinnitus and reduce tinnitus-related distress (Attarha et al., 2018; Hobson et al., 2012). Li et al. (2016) reported that 12 months of personalised music therapy was associated with reduced tinnitus-related distress and improvement in selected patient-reported outcomes. However, Langguth et al. and Henry et al. noted that the evidence base for sound therapy in chronic tinnitus remains limited, and that further large-scale randomized controlled trials are needed to clarify its long-term effectiveness and safety (Henry, 2022; Langguth et al., 2013). Photobiomodulation has been proposed as a light-based intervention that may influence inflammatory processes or neural repair, although its mechanisms remain incompletely understood. Choi et al. (2024) reported that photobiomodulation was associated with improvement in tinnitus-related distress and psychological burden. However, Talluri et al. (2022) noted that most photobiomodulation studies did not demonstrate significant benefit, with small sample sizes and inconsistent outcome measures further limiting comparability and reproducibility. Electrical stimulation has also been investigated as a possible intervention in chronic tinnitus, but its clinical value remains uncertain, and further well-designed studies are needed to clarify its long-term relevance and stability (Chen et al., 2023). Overall, rehabilitation approaches may benefit some patients, particularly in reducing tinnitus-related distress or supporting coping; however, their broader clinical use remains limited by heterogeneous treatment response, insufficient long-term evidence, and the lack of standardized protocols.

### 2.3 Psychological treatment for chronic tinnitus

Psychological interventions for chronic tinnitus aim to modify patients' cognitive, emotional, and behavioral responses to tinnitus rather than directly reducing the perceived sound itself. In clinical practice, these approaches primarily target tinnitus-related distress, mood-related symptoms, sleep disturbance, and patients' ability to cope with tinnitus in daily life. Common psychological interventions include cognitive behavioral therapy (CBT), relaxation-based approaches, tinnitus retraining therapy (TRT), and mindfulness-based interventions (McFerran et al., 2019).

Among these, CBT is the best-established psychological treatment for chronic tinnitus and is widely regarded as the current gold standard. It is intended to reduce tinnitus-related distress by helping patients identify and modify maladaptive thoughts and behaviours (Fuller et al., 2020). Available evidence suggests that CBT may improve tinnitus-related distress and associated emotional outcomes in some patients (Cima et al., 2012). Beukes et al. (2022) reported that CBT may reduce the psychological burden associated with tinnitus by targeting maladaptive cognitive and behavioral responses. Similarly, Li et al. (2019) found that CBT was associated with reductions in tinnitus-related distress and improvements in mood-related symptoms and stress. Marks et al. (2022) further reported that insomnia-focused cognitive behavioral therapy (CBTi) was associated with greater improvement than traditional audiological care and sleep support therapy in tinnitus-related distress, sleep quality, and mental health outcomes; however, these findings should be interpreted with appropriate caution (McKenna et al., 2014). Reported benefits appear to relate mainly to tinnitus-related distress, sleep, and emotional functioning rather than to direct reductions in tinnitus loudness. However, variation in treatment protocols, patient adherence, comparator conditions, and outcome measures complicates interpretation of the consistency and durability of benefit across clinical settings. Baguley et al. (2013) noted that the application of CBT in tinnitus management faces several challenges, including variation in treatment protocols, differences in patient adherence, and uncertainty regarding long-term effects. Aazh et al. (2019) likewise suggested that, although CBT appears beneficial for some patients, its comparative efficacy relative to other psychological interventions has not been fully established. Fuller et al. (2020) further emphasized that heterogeneity in outcome measures and the lack of standardized evaluation criteria contribute to ongoing uncertainty regarding long-term effectiveness. Overall, CBT remains a central psychological approach in chronic tinnitus management, but its implementation and reported effects are influenced by variability in delivery, patient engagement, and limitations in the current evidence base, particularly with respect to long-term follow-up and comparability across studies (McKenna et al., 2020).

Relaxation-based interventions, often incorporated into broader behavioral programmes, are intended to reduce physical tension, anxiety, and stress, and may thereby lessen tinnitus-related distress in some patients (Fuller et al., 2020). In tinnitus management, this approach may be helpful for stress reduction and emotional regulation. Weber et al. (2002); Thompson et al. (2017) reported that relaxation training may reduce the influence of negative emotional states on tinnitus perception by alleviating tinnitus-related anxiety and stress. However, Ireland et al. (1985) found that its effects in chronic tinnitus were limited, with significant improvement observed only in depression scores and not in other measured outcomes. Overall, relaxation training may be helpful for stress management and emotional regulation in some tinnitus patients, but the current evidence remains limited and requires further validation.

Tinnitus retraining therapy (TRT) is designed to help patients adapt to tinnitus and reduce negative emotional responses through a combination of counseling and sound-based strategies. Its theoretical basis is to weaken the negative associations between tinnitus and the auditory, limbic, and autonomic nervous systems, thereby reducing tinnitus-related distress (Alashram, 2024). Phillips and McFerran (2010) reported that TRT was associated with greater improvement in tinnitus-related distress than tinnitus masking treatment. Luyten et al. (2020) also found that combining TRT with CBT was associated with improvements in tinnitus-related distress and related patient-reported outcomes, with effects remaining stable after 3 months. However, Han et al. (2021) noted in a meta-analysis that although TRT may provide short-term benefit, the available studies are affected by methodological weaknesses and risk of bias, limiting confidence in the conclusions. In addition, some studies have reported no significant difference between TRT and standard care (Tinnitus Retraining Therapy Trial Research et al., 2019). Suh et al. (2023) also found that combining TRT with CBT was associated with improvements in tinnitus-related distress and related patient-reported outcomes, with effects remaining stable after 3 months. Overall, TRT may help some patients adapt to tinnitus and reduce tinnitus-related distress, but its clinical value remains constrained by methodological limitations in the evidence base, treatment burden, and uncertainty regarding long-term benefit.

Taken together, the psychological approaches described above may provide benefit for tinnitus-related distress, but each also has important limitations, including treatment burden, variability in response, and uncertainty regarding long-term outcomes. Against this background, mindfulness-based interventions, which emphasize non-judgmental awareness of present-moment experiences, have received increasing attention as a potential psychological approach for chronic tinnitus. Rather than attempting to reduce the perceived sound itself, mindfulness-based interventions aim to change how patients relate to tinnitus by improving emotional regulation, acceptance, and coping. Existing studies suggest that these approaches may help reduce tinnitus-related distress and improve quality of life (Derlic, 2022).

## 3 Mindfulness-based therapy

### 3.1 Overview of mindfulness-based therapy

Mindfulness-based therapy is a psychological approach centered on present-moment awareness and a non-judgemental, accepting attitude toward internal and external experiences (Alvear et al., 2022; Kiken et al., 2015). In clinical practice, this approach encourages individuals to notice thoughts, emotions, and bodily sensations as they arise, without immediately reacting to them or attempting to suppress or avoid them. In chronic tinnitus, mindfulness-based therapy is not intended to alter the auditory percept itself or to reduce tinnitus loudness directly. Rather, its therapeutic focus is to modify how patients attend to, interpret, and emotionally respond to the tinnitus percept. Core elements of mindfulness-based therapy include sustained present-focused attention, acceptance of ongoing experience, enhanced awareness of bodily and emotional states, and reduced reactivity to distressing internal events. These skills are typically developed through structured practices such as mindful breathing, body scan exercises, and meditation (Goldberg et al., 2018). When applied to chronic tinnitus, such practices may help patients reduce habitual monitoring of tinnitus, weaken maladaptive cognitive-emotional responses, and develop a more adaptive relationship with the condition. Although mindfulness-based approaches have been applied in a range of clinical settings, their role in chronic tinnitus should be interpreted with conceptual precision. Current evidence primarily supports their use in reducing tinnitus-related distress, improving emotional well-being, and enhancing quality of life, rather than in directly decreasing tinnitus loudness.

### 3.2 Potential clinical features of mindfulness-based therapy

Mindfulness-based therapy is a non-pharmacological approach that has been applied in the management of chronic conditions, including chronic tinnitus. In this setting, its potential clinical value lies not in altering the tinnitus percept itself, but in helping patients modify their cognitive, attentional, and emotional responses to tinnitus. By encouraging a more accepting and less reactive stance toward the tinnitus experience, mindfulness-based interventions may help reduce tinnitus-related distress and improve psychological well being (Lee and Jung, 2023).

Compared with pharmacological approaches, which may provide symptom relief in some patients but are often limited in long-term effectiveness (Coles, 1987), mindfulness-based therapy aims to support more adaptive coping through repeated attentional and emotional regulation practices. In patients with chronic tinnitus, this may help reduce maladaptive monitoring of the tinnitus percept and lessen associated anxiety or depressive symptoms (Kim et al., 2017). However, these potential benefits should be interpreted mainly in relation to tinnitus-related distress and broader quality-of-life outcomes, rather than as evidence of a direct reduction in tinnitus loudness (Thompson et al., 2017).

Another potential feature of mindfulness-based therapy is its flexibility in delivery and practice intensity. Intervention duration, session frequency, and home practice requirements may be adjusted according to individual needs, clinical context, and patient engagement. This personalized and participatory format may enhance the feasibility of long-term management for some patients, although sustained adherence can remain challenging in practice.

Mindfulness-based therapy may also be delivered through digital platforms, which could improve accessibility and support remote intervention. Recent work has explored technology-assisted formats, including app-based and digitally supported mindfulness programmes. For example, Kora et al. (2021) described an AI-assisted meditation feedback system incorporating deep learning and neurophysiological analysis. However, such applications should currently be regarded as preliminary and exploratory, and further evidence is needed to determine their clinical value, feasibility, and added benefit in tinnitus management.

### 3.3 Clinical applications of mindfulness-based therapy

Mindfulness-based therapy has been applied across a range of clinical conditions, including anxiety, depression, chronic pain, cardiovascular disorders, and autism spectrum disorder. Evidence from these fields suggests that mindfulness-based interventions may support emotional regulation, reduce perceived stress, and improve aspects of quality of life. In the context of chronic tinnitus, findings from these broader clinical applications may provide a useful conceptual framework for understanding how mindfulness-based approaches could help patients cope more adaptively with tinnitus-related distress (Beerse et al., 2020; Janssen et al., 2018; Liu et al., 2021; Schuman-Olivier et al., 2020). However, such evidence should be interpreted cautiously, as findings from other clinical populations cannot be assumed to translate directly to tinnitus.

In the treatment of anxiety disorders, Evans et al. (2008) reported that mindfulness-based therapy was associated with improvement in anxiety symptoms, and Miller et al. (1995) suggested that some of these effects may persist for up to 3 months. A review by Segal and Walsh (2016) further indicated that mindfulness-based approaches may also be associated with improvements in depressive symptoms and sleep-related outcomes in some populations. These findings are potentially relevant because anxiety, depression, and sleep disturbance frequently coexist with chronic tinnitus, but they should not be interpreted as direct evidence of tinnitus-specific benefit. A similar point applies to chronic pain research, Mindfulness-based interventions have been reported to improve pain-related outcomes and to help patients respond more adaptively to persistent symptoms, thereby reducing their impact on daily life. Hilton et al. (2016) reported in a meta-analysis that mindfulness-based therapy was associated with changes in pain-related outcomes, while Cherkin et al. (2016) suggested that these interventions may help patients respond more adaptively to persistent symptoms and reduce the impact of those symptoms on quality of life. This literature may offer a useful conceptual parallel for tinnitus, as both conditions can involve persistent internal sensations that are shaped by attention, interpretation, and emotional reactivity. Even so, such comparisons remain indirect, and the underlying mechanisms and outcome domains should not be treated as equivalent. In cardiovascular settings, mindfulness-based interventions have been investigated as supportive strategies for stress reduction and behavioral self-regulation, with some studies reporting modest benefits in blood pressure and postoperative quality of life (Kubzansky et al., 2018; Loucks et al., 2015). These findings may be relevant to the broader role of mindfulness in reducing stress-related burden, but they provide only limited indirect support for its relevance to tinnitus. Additionally, mindfulness-based therapy has shown promising prospects in emotional regulation for children and adolescents with autism spectrum disorder (Semple, 2019). Research indicates that mindfulness-based interventions are particularly effective in improving emotional regulation in adolescents with autism (Beck et al., 2020).

## 4 Application of mindfulness-based therapy in chronic tinnitus treatment

### 4.1 Evolution and innovation of mindfulness-based therapy in tinnitus treatment

Since the introduction of Mindfulness-Based Stress Reduction (MBSR) by Kabat-Zinn in 1979, mindfulness-based approaches have gradually been extended to a range of chronic conditions, including tinnitus (Morgan, 2003; Shapero et al., 2018). As shown in Table 1, studies of mindfulness-based interventions in chronic tinnitus began to emerge in the mid-2000s and have since developed from early exploratory applications to more structured psychological programmes, particularly Mindfulness-Based Cognitive Therapy (MBCT). Across this literature, mindfulness-based approaches have generally been considered as potential interventions for reducing tinnitus-related distress and improving coping, rather than for directly reducing tinnitus loudness.

**Table 1:** Research on mindfulness-based therapy in chronic tinnitus treatment in recent years.

References & Year	Follow up	Tinnitus duration	Intervention	Comparator	Main outcome measures/domains
Mazurek et al. (2005)	6 months	6	MBCT	Relaxation therapy	TQ (tinnitus-related distress): 49.2 vs. 60.2 HADS-A (anxiety): 10.2 ± 3.7 vs. 11.0 ± 4.2 HADS-D (depression): 7.5 ± 4.2 vs. 8.5 ± 4.0
Sadlier et al. (2007)	4–6 months	6	MBCT and mindfulness meditation	Waiting list	HADS-A (anxiety): 7.9 vs. 9.4 HADS-D (depression): 3.9 vs. 4.8
Biesinger et al. (2010)	1–3 months	3	Mindfulness-based Qigong	Waiting list	TBF-12 (tinnitus-related burden): 8.0 ± 3.0 vs. 13.3 ± 4.1 VAS (self-reported tinnitus-related burden; construct may vary by study): 4.3 ± 1.3 vs. 6.6 ± 1.7
Philippot et al. (2011)	3 months	6	MBCT	Relaxation	BDI (depression): 9.38 ± 10.44 vs. 11.92 ± 7.23 STAI (anxiety/state-trait anxiety): 40.46 ± 12.37 vs. 45.73 ± 10.47
Hesser et al. (2012)	1 year	6	Mindfulness-based and body-oriented group therapy	Waiting list	BDI (depression): 9.00 ± 5.66 vs. 12.83 ± 5.36 STAI (anxiety): 39.84 ± 9.76 vs. 45.75 ±7.72
Hesser et al. (2012)	6 months	6	Mindfulness meditation	Documentary Group	THI (tinnitus-related distress/handicap): 31.94 ± 14.54 vs. 49.94 ± 16.09 HADS-A (anxiety): 4.21 ± 2.25 vs. 6.78 ± 3.98 HADS-D (depression): 3.48 ± 2.43 vs. 4.59 ± 3.29
Roland et al. (2015)	1 year	6	MBSR	No control	THI (tinnitus-related distress/handicap):14 (range:4–32) vs. 28 (range:20–50) TFI (tinnitus-related functional impact): 23 (range:4–48) vs. 39 (range: 24–61)
Gao and He (2015)	6 months	6	ACT	TRT	THI (tinnitus-related distress/handicap): 28.54 ± 18.18 vs. 42.15 ± 19.22 HADS-A (anxiety): 3.22 ± 3.41 vs. 5.89 ± 4.33 HADS-D (depression): 3.71 ± 3.23 vs. 7.09 ± 4.32
Arif et al. (2017)	6 months	6	Mindfulness meditation	Relaxation therapy	TRQ (tinnitus-related distress/reaction): 15.06 ± 13.12 vs. 19.59 ± 13.15 HADS-A (anxiety): 4.59 ± 2.80 vs. 5.89 ± 4.02 HADS-D (depression): 4.82 ± 2.96 vs. 5.15 ± 3.78
McKenna et al. (2017)	6 weeks	6	MBCT	Relaxation therapy	TQ (tinnitus-related distress): 30.44 ± 1.21 vs. 42.52 ± 1.11 CORE-NR (general psychological distress): 11.80 ± 0.58 vs. 15.61 ± 0.56
Gans et al. (2023)	6 months	6	i-MBTSR	Delayed treatment group	TFI (tinnitus-related functional impact): 34.23 ± 2.91 vs. 59.96 ± 2.61 PSS (perceived stress): 17.73 ± 1.11 vs. 26.41 ± 1.29
Walter et al. (2023)	3 months	6	Kalmeda app and CBT	Control group	TQ (change in tinnitus-related distress): −13.36 ± 1.00 vs. −0.63 ± 0.98 PHQ-9 (change in depressive symptoms): −1.3 ± 2.4 vs. + 0.3 ± 2.4 PSQ-20 (change in perceived stress): −4.2 ± 11.9 vs. −0.5 ± 9.5

ACT, acceptance and commitment therapy; BDI, beck depression inventory; CORE-NR: clinical outcomes in routine evaluation—non-risk items; HADS-A, hospital anxiety and depression scale—anxiety subscale; HADS-D, hospital anxiety and depression scale—depression subscale; i-MBTSR, internet-based mindfulness-based tinnitus stress reduction; Kalmeda app, a smartphone-based cognitive behavioral therapy (CBT) app designed for managing chronic tinnitus; MBCT, mindfulness-based cognitive therapy; MBSR, mindfulness-based stress reduction; PHQ-9, patient health questionnaire-9; PROMIS, patient-reported outcomes measurement information system; PSQ-20, psychosocial questionnaire-20; PSS, perceived stress scale; RCT, randomized controlled trial; SCL-90, symptom checklist-90; STAI, state and trait anxiety inventory; THI, tinnitus handicap inventory; TQI, tinnitus questionnaire index; TRT, tinnitus retraining therapy; TRQ, tinnitus reaction questionnaire; VAS, visual analog scale; VAS-Distress, visual analog scale for distress

Early exploratory studies suggested that mindfulness meditation might improve tinnitus-related distress and associated emotional symptoms in some patients with chronic tinnitus. For example, Mazurek et al. (2005) reported early clinical observations supporting the potential relevance of mindfulness-based approaches in tinnitus care. However, the mechanisms proposed in this early work, such as neuroplastic modulation or sensory desensitization, should be interpreted cautiously, as direct mechanistic evidence remains limited. Similarly, Arif et al. (2017) reported that mindfulness meditation was associated with greater improvement in anxiety and depression symptoms than relaxation therapy in tinnitus patients, while Philippot et al. (2011) described potential longer-term benefits for psychological distress. These findings are of interest, but they primarily relate to emotional distress and coping rather than direct changes in tinnitus loudness.

Subsequent research increasingly focused on more structured mindfulness-based programmes, particularly Mindfulness-Based Cognitive Therapy (MBCT). Mazurek et al. (2005) innovatively proposed the shift from traditional mindfulness-based interventions (MBI) to structured mindfulness-based cognitive therapy (MBCT). Some studies have suggested that MBCT may reduce tinnitus-related distress and improve associated emotional symptoms in patients with chronic tinnitus (McKenna et al., 2018; Sadlier et al., 2007). These findings have contributed to growing clinical interest in mindfulness-based approaches. Nevertheless, the evidence remains heterogeneous, with substantial variation in study design, comparator conditions, follow-up duration, and outcome measures. As a result, the current literature does not support broad conclusions regarding consistent efficacy across all patient populations or outcome domains.

Combined or integrative treatment approaches have also been explored in tinnitus care. In broader mental health settings, mindfulness-based therapy has been combined with a range of interventions, and similar approaches have been investigated in tinnitus (Britton et al., 2012; Fjorback et al., 2011). Such approaches may be of interest because chronic tinnitus often involves cognitive, emotional, attentional, and behavioral dimensions. However, the available evidence remains limited, and direct comparisons with single-modality interventions are not yet sufficient to support broad conclusions regarding superiority. For example, Kreuzer et al. (2012) described a combined intervention incorporating mindfulness training and somatic-psychological therapy and reported improvement in some patient-reported outcomes. However, because this type of intervention includes multiple therapeutic components, it is difficult to determine the specific contribution of mindfulness-based elements. Hesser et al. (2012) also examined approaches incorporating mindfulness, Acceptance and Commitment Therapy (ACT), and Cognitive Behavioural Therapy (CBT). These integrative formats may expand the range of psychological strategies available in tinnitus management, but the evidence supporting them remains preliminary and heterogeneous.

Mindfulness-based therapy has also been explored in relation to CBT and other psychotherapeutic approaches, although the evidence supporting such integration remains limited. In psychological research more broadly, mindfulness-based elements have been combined with cognitive and behavioural strategies to address maladaptive thought patterns, emotional reactivity, and avoidance (Cladder-Micus et al., 2018; Wolever et al., 2022). In chronic tinnitus, MBCT has received particular attention, and selected studies suggest possible benefit in tinnitus-related distress and related psychological outcomes following MBCT-based intervention (McKenna et al., 2017). However, these findings should be interpreted cautiously, as the reported outcomes relate primarily to distress-related and psychological domains rather than to direct changes in tinnitus loudness. Other forms of psychotherapeutic integration, including combinations with solution-focused, psychodynamic, or somatic approaches, have also been described (Churchill, 2022; Hu et al., 2024; Mehling et al., 2011; Price et al., 2007). These models may be relevant in selected clinical contexts or patient subgroups. However, the evidence supporting their application in chronic tinnitus remains limited, and in many cases the discussion is more theoretical or exploratory than supported by robust clinical evidence. Accordingly, these approaches are best regarded as adjunctive or investigational rather than established treatment models.

Interdisciplinary approaches involving music therapy or physical therapy have also been explored in chronic tinnitus. For example, Feng et al. (2020) combined music therapy with cognitive behavioral therapy and incorporated mindfulness exercises in patients with tinnitus. EEG analysis indicated significant increases in α and θ wave power following the intervention. However, as EEG is an indirect measure, these findings should be interpreted cautiously and may reflect changes in tinnitus-related distress, attentional processing, or emotional reactivity rather than definitive evidence of a specific therapeutic mechanism or direct reduction in tinnitus loudness. Similarly, Michiels (2023) reviewed the combined use of mindfulness-based therapy and physical therapy, such as cervical muscle relaxation training, particularly in subgroups of patients with possible somatic or cervicogenic contributions to tinnitus. As with other multimodal approaches, however, the specific contribution of the mindfulness-based component remains difficult to isolate. Although such approaches may be relevant for selected patients, the currently available evidence remains limited and is insufficient to support broad generalization.

Some studies have also explored possible neurobiological correlates of mindfulness-based therapy in tinnitus. Roland et al. (2015) used resting-state functional connectivity MRI to examine changes in attention-related brain networks following mindfulness-based intervention, while Husain et al. (2019) used functional magnetic resonance imaging (fMRI) and electroencephalography (EEG) to investigate possible structural or functional brain changes associated with mindfulness practice. These studies provide preliminary observations that may help generate hypotheses regarding attention, emotion regulation, and cognitive processing in tinnitus. However, such findings should not be interpreted as establishing a definitive neurobiological mechanism of action. Neuroimaging changes do not in themselves demonstrate causal pathways, and the clinical significance of these observations remains unclear. Future work may benefit from further multimodal investigation, but at present this area should be regarded as exploratory rather than established.

To improve accessibility, mindfulness-based approaches have also been adapted to digital and online formats. Hesser et al. (2012) examined an internet-delivered intervention incorporating ACT and CBT components, while Chatterjee et al. (2021) and Gans et al. (2023) reported app-based or online mindfulness-related interventions associated with improvements in tinnitus-related distress or perceived stress. Li-Jessen et al. (2023) also described a digital intervention combining mindfulness-based elements with CBT. These studies suggest that remote delivery may be feasible and potentially beneficial for some patients, particularly in relation to accessibility and support for self-management. However, the evidence remains limited by heterogeneity in intervention content, comparator conditions, follow-up duration, and outcome definitions. Therefore, digital and app-based approaches should currently be regarded as promising but still developing formats rather than well-established interventions.

In conclusion, mindfulness-based therapy has received increasing attention as a psychological approach in chronic tinnitus care. From earlier mindfulness-based interventions to the development of Mindfulness-Based Cognitive Therapy (MBCT) and its integration with other therapeutic approaches, such as ACT and CBT, this field has evolved toward more structured and clinically oriented applications. However, the available evidence remains heterogeneous, and current findings suggest potential benefit primarily in tinnitus-related distress and associated psychological burden rather than in direct reduction of tinnitus loudness. Neurobiological research and digital interventions may offer additional perspectives for understanding and delivering mindfulness-based therapy in chronic tinnitus, but the current evidence remains preliminary and should not be interpreted as providing definitive validation of treatment mechanisms or broader clinical effectiveness. At present, mindfulness-based therapy is better understood as an approach that may reduce tinnitus-related distress and associated psychological burden through changes in attention, appraisal, and coping, rather than as an intervention that directly reduces tinnitus loudness. Its acceptability and potential benefit may vary across patients and clinical contexts. Future research may explore whether mindfulness-based therapy can be integrated with other approaches, including neuromodulatory strategies, but such possibilities remain exploratory and require stronger empirical support and a clear clinical or mechanistic rationale.

## 5 Efficacy assessment of mindfulness-based therapy in chronic tinnitus treatment

### 5.1 Efficacy research of mindfulness-based therapy

As summarized in Table 2, several randomized controlled trials and review-based studies have examined mindfulness-based interventions in chronic tinnitus. Overall, the available evidence suggests that these interventions may improve certain patient-reported outcomes, particularly tinnitus-related distress, psychological distress, and associated emotional or cognitive responses. However, the findings should be interpreted cautiously because the included studies differ substantially in intervention format, comparator condition, sample size, follow-up duration, and outcome measures. Importantly, the reported benefits relate primarily to tinnitus-related distress and broader psychological functioning, rather than to direct reductions in tinnitus loudness.

**Table 2:** Summary of efficacy of mindfulness-based therapy in chronic tinnitus treatment.

Article	Study characteristics	Results	Strengths	Limitations
Sadlier et al. (2007)	Intervention: cognitive behavioral therapy/meditation Control group: waiting list Sample size: *n* = 25 Average age: not available	Feasibility and acceptability were reported as favorable. For the primary outcome of tinnitus-related distress, both groups showed reduction, with no statistically significant between-group difference. Secondary outcomes suggested improvement in some distress-related or disability-related domains in the mindfulness-based group (*p* < 0.05).	Standardized intervention program Participants randomized and blinded to control group assignment Primary and secondary outcomes clearly defined	Small sample size (pilot study), underpowered No active control group
Philippot et al. (2011)	Intervention: mindfulness therapy Control group: control group Sample size: unknown Average age: not available	Reduction in tinnitus-related distress was reported (*p* < 0.05). Secondary outcomes also suggested improvement in emotional regulation and mindfulness-related measures (*p* < 0.05).	Well-defined intervention Primary and secondary outcomes clearly defined	Small sample size, unclear randomization process No control group
Rademaker et al. (2019)	Intervention: mindfulness-based interventions Control group: varied (RCTs, cohort studies) Sample size: *n* = 425 Average age: not available	Pooled findings suggested a possible beneficial effect on tinnitus-related distress (*p* < 0.01). Some included studies also reported sustained improvement in distress-related outcomes over follow-up; however, interpretation is limited by heterogeneity in study design, intervention type, and outcome measures.	Large sample size Varied studies and robust meta-analysis	Mixed sample design, findings may vary based on study quality Limited long-term data
Kreuzer et al. (2012)	Intervention: mindfulness and body-psychotherapy-based intervention Control group: waiting list Sample size: *n* = 36 Average age: not available	Improvement in tinnitus-related distress was reported (*p* < 0.05), together with improvement in self-regulation-related outcomes (*p* < 0.05). Because the intervention was multimodal, the specific contribution of mindfulness-based components remains difficult to isolate.	Comprehensive intervention program Clear study design and randomization	Small sample size, underpowered No active control group
McKenna et al. (2018)	Intervention: MBCT (mindfulness-based cognitive therapy) Control group: none Sample size: *n* = 182 Average age: not available	Improvement in tinnitus-related distress and general psychological distress was reported after treatment (*p* < 0.05). These outcomes should be interpreted primarily as reductions in distress-related burden rather than direct reductions in tinnitus loudness. The study used a clear treatment protocol, but interpretation is limited by the absence of an active control group and long-term follow-up.	Large sample size Clear treatment protocol with consistent findings	No active control group No long-term follow-up
Husain et al. (2019)	Intervention: adjusted MBCT Control group: none Sample Size: *n* = 15 Average age: not available	Reduction in tinnitus-related distress was reported (*p* < 0.05). Exploratory secondary findings suggested structural neuroimaging changes following intervention; however, these observations should be regarded as preliminary and should not be interpreted as direct evidence of tinnitus reduction or definitive mechanism.	Structural MRI analysis supporting the intervention effects Clear study design	Small sample size, underpowered No active control group
Marks et al. (2019)	Intervention: MBCT for tinnitus Control group: none Sample Size: *n* = 9 Average age: not available	Reduction in tinnitus-related distress was reported (*p* < 0.05), together with improvement in emotional and cognitive response domains post-treatment (*p* < 0.01). These findings are supportive but preliminary, given the small sample size and lack of long-term follow-up.	Well-defined intervention and clear outcome measures Explored patients' direct experiences	Small sample size, underpowered No long-term follow-up

MBCT, mindfulness-based cognitive therapy; MBSR, mindfulness-based stress reduction; MRI, magnetic resonance imaging.

Sadlier et al. (2007) reported that, following a combined intervention involving cognitive behavioral therapy and meditation, patients with chronic tinnitus showed improvement in psychological variables such as pessimism and acceptance (*P* = 0.001; *P* = 0.002). Visual Analog Scale (VAS) scores also decreased (*P* = 0.007), although the specific construct assessed by VAS may vary across studies. In addition, 80% of patients reported continued improvement in self-reported tinnitus-related distress or symptom burden during follow-up at 4–6 months. However, these findings should be interpreted in relation to psychological adaptation and self-reported burden rather than as evidence of direct change in the tinnitus percept itself. Philippot et al. (2011) found that mindfulness-based treatment was associated with improvement in negative emotional states, anger, acceptance, rumination, and emotional regulation, and acceptance in patients with chronic tinnitus. After 3 months of follow-up, the mindfulness group still showed significant improvements in emotional regulation, cognitive processing, and reduction in rumination. These findings suggest that mindfulness-based therapy may help patients respond differently to tinnitus at the emotional and cognitive levels. However, they do not indicate that the intervention directly reduced tinnitus loudness, and interpretation is limited by the size and design of the study. Additionally, a meta-analysis by Rademaker et al. (2019) further suggested that mindfulness-based interventions may reduce tinnitus-related distress. However, the interpretation of pooled findings requires caution because the included studies varied in intervention type, comparator condition, outcome definition, and methodological quality. Accordingly, the meta-analytic evidence may support a possible beneficial effect on tinnitus-related distress, but it does not establish uniform efficacy across all mindfulness-based approaches or patient groups. Some studies have also reported maintenance of benefit over follow-up periods. For example, McKenna et al. (2017) and Gans et al. (2015) described improvements that were sustained for up to 6 months. While these findings are encouraging, longer-term outcomes remain insufficiently characterized, and the durability of benefit may vary according to intervention structure, adherence, and patient characteristics. Kreuzer et al. (2012) reported that patients receiving mindfulness-based and somatic-psychological therapy showed improvement in Tinnitus Questionnaire scores (*P* < 0.001), with the mindfulness-based intervention performing better than the comparison condition on some measures (*P* = 0.023). However, because this type of study involves complex or multimodal treatment designs, it may be difficult to isolate the specific contribution of mindfulness-based components. McKenna et al. (2018) evaluated an 8-week Mindfulness-Based Cognitive Therapy intervention in 182 patients with chronic tinnitus and reported clinically relevant improvement in tinnitus-related distress in 50% of participants and in psychological distress in 41.2%. These findings provide relatively stronger support for the potential benefit of MBCT than smaller preliminary studies. Nevertheless, the reported outcomes should be interpreted as reflecting improvement in distress-related and psychological domains rather than direct reduction in tinnitus loudness. Husain et al. (2019) used an modified MBCT to treat tinnitus-related distress, with results showing significant reductions in tinnitus distress and a significant increase in gray matter volume in the bilateral superior frontal gyrus. Used an modified MBCT to treat tinnitus-related distress, with results showing significant reductions in tinnitus distress and a significant increase in gray matter volume in the bilateral superior frontal gyrus. Although these neuroimaging observations are of mechanistic interest, they remain preliminary and should not be interpreted as establishing a definitive neurobiological pathway through which mindfulness-based therapy exerts its effects. The clinical significance of these imaging findings also remains uncertain. Marks et al. (2019) described sustained improvement in tinnitus-related distress and emotional-cognitive outcomes after MBCT, but the very small follow-up sample substantially limits generalisability. Accordingly, such findings should be regarded as supportive but preliminary rather than definitive evidence of long-term effectiveness.

Taken together, the current literature suggests that mindfulness-based therapy may benefit some patients with chronic tinnitus, particularly by reducing tinnitus-related distress and improving emotional or cognitive coping. However, the evidence base remains heterogeneous, and the available studies are not directly comparable with respect to intervention content, control conditions, follow-up duration, or outcome constructs. Therefore, conclusions regarding overall efficacy should remain cautious. Future studies should adopt clearer classification of outcome domains, larger sample sizes, active control groups, and longer follow-up in order to define more precisely the magnitude, durability, and scope of benefit.

### 5.2 Comparison of efficacy between mindfulness-based therapy and other treatments

As shown in Table 3, several studies have compared mindfulness-based interventions with other psychological or behavioral approaches, including relaxation-based interventions, in patients with chronic tinnitus. Overall, these comparisons suggest that mindfulness-based therapy may offer benefits for some patient-reported outcomes, particularly tinnitus-related distress, psychological distress, emotional functioning, and tinnitus acceptance. However, these findings should be interpreted cautiously because the comparator conditions, intervention formats, and outcome measures differ across studies. In addition, the available comparisons relate mainly to distress-related and psychological outcomes rather than to direct changes in tinnitus loudness. McKenna et al. (2017) reported that, compared with relaxation therapy, Mindfulness-Based Cognitive Therapy (MBCT) was associated with greater improvement in tinnitus-related distress and psychological distress, with benefits maintained at 1-month and 6-month follow-up. Arif et al. (2017) found that the mindfulness meditation group showed a reduction in Tinnitus Reaction Questionnaire (TRQ) scores (*P* = 0.047) compared to the relaxation therapy group, suggesting that mindfulness meditation may be more effective than relaxation therapy in reducing tinnitus-related distress, anxiety, and depression. Philippot et al. (2011) also reported that mindfulness meditation was associated with greater improvement than relaxation training in negative emotions, rumination, social functioning, and tinnitus acceptance (*P* < 0.05). Together, these studies suggest that mindfulness-based approaches may be particularly relevant for modifying maladaptive emotional and cognitive responses to tinnitus (*P* < 0.05). By contrast, Beukes et al. (2021) found that, compared to relaxation therapy, internet-based cognitive behavioral therapy (ICBT) showed improvements in tinnitus cognition at both the end of the intervention and during follow-up (*P* < 0.05). de Fockert et al. (2013) further noted that under mindfulness intervention, the negative impact of suppression strategies on task persistence was significantly alleviated, suggesting that mindfulness intervention can effectively mitigate the negative effects of suppressing emotional negative sounds. Arancibia and Papuzinski (2022) reviewed various psychological treatment methods for tinnitus and found that MBSR and MBCT had significant advantages in treating tinnitus-related symptoms and psychological distress.

**Table 3:** Comparison of efficacy between mindfulness-based therapy (MBT) and other psychological therapies in tinnitus treatment.

References	Study design	Sample size	Follow-up	Intervention	Comparator	Main outcome measures	Summary of findings
McKenna et al. (2017)	RCT	75	1.6 M	MBCT	RT	THI, HADS-A, HADS-D	MBCT was associated with greater improvement than RT in THI-defined tinnitus-related distress/handicap, with a mean difference of 6.3 (95% CI 1.3–11.4, p = 0.016). This effect was maintained at 6-month follow-up, with a mean difference of 7.2 (95% CI 2.1–12.3, *p* = 0.006), corresponding to a standardized effect size of 0.56 (95% CI 0.16–0.96). These findings support possible benefit primarily in tinnitus-related distress rather than in tinnitus loudness.
Arif et al. (2017)	RCT	86	NR	MBI	RT	TQ, TRQ, HADS-A, HADS-D	Compared with relaxation therapy, mindfulness-based intervention was associated with improvement in tinnitus-related distress (TQ/TRQ) and in anxiety- and depression-related outcomes (HADS-A/HADS-D). Reported effect sizes ranged from moderate to large. However, these findings relate mainly to tinnitus-related distress and psychological burden, rather than to direct changes in tinnitus loudness.
Philippot et al. (2011)	RCT	25	3	MBCT RT	Psychoeducation	QIPA, BDI, STAI	The mindfulness group showed greater improvement at follow-up in negative emotional states, rumination, frustration, and acceptance-related outcomes than the comparison group. These findings suggest possible benefit in emotional and cognitive response domains, although the small sample size and limited follow-up warrant cautious interpretation.
Beukes et al. (2021)	RCT	126	2	ICBT	Applied relaxation	Tinnitus functional index, anxiety, depression, Insomnia	Both internet-based CBT and applied relaxation were associated with improvement in tinnitus-related functional burden, with somewhat greater improvement reported for internet-based CBT. The between-group difference was small. These findings suggest that different psychological approaches may improve overlapping but not identical outcome domains.
Hesser et al. (2012)	RCT	99	1 year	ACT/CBT	forum	THI, HADS-A, PSS, QoL	Both ACT and CBT were associated with improvement in THI-defined tinnitus-related distress/handicap, with broadly similar effects across groups. Additional improvement was reported in anxiety, perceived stress, and quality-of-life-related outcomes. These findings support possible benefit across several distress-related domains, although the effects were not specific to mindfulness-based intervention alone.
Arancibia and Papuzinski (2022)	Review	Varied	Varied	CBT, mindfulness-based Stress reduction, acceptance	Various (including CBT)	Distress, quality of life, severity, depression	The review suggested that CBT- and mindfulness-based approaches may both be beneficial for tinnitus-related distress, disability-related burden, and quality-of-life-related outcomes. CBT appeared more closely associated with change in negative cognitions, whereas mindfulness-based approaches may be particularly relevant to acceptance, positive coping, and distress reduction. However, comparisons across studies were limited by heterogeneity in intervention type, comparator conditions, and outcome definitions.

ACT, acceptance and commitment therapy; BDI, beck depression inventory; CBT, cognitive behavioral therapy; RT, relaxation therapy; HADS-A: hospital anxiety and depression scale—anxiety; HADS-D, hospital anxiety and depression scale—depression; ICBT, internet-based cognitive behavioral therapy; MBCT, mindfulness-based cognitive therapy; MBI, mindfulness-based intervention; THI, tinnitus handicap inventory; TQ, tinnitus questionnaire; TRQ, tinnitus reaction questionnaire; N, sample size; PSS, perceived stress scale; QIPA, questionnaire on interpersonal problems assessment; QoL, quality of life; TRQ, tinnitus reaction questionnaire

## 6 Limitations and future research directions

### 6.1 Limitations

Although mindfulness-based therapy may benefit some patients with chronic tinnitus, several limitations should be recognized. Its effects appear to relate mainly to reductions in tinnitus-related distress and associated psychological burden, rather than to direct reductions in tinnitus loudness. In addition, this approach requires sustained engagement and regular practice, which may be difficult for some patients and may contribute to reduced adherence or drop-out. Some individuals may also find early mindfulness practice challenging or temporarily distressing. Mindfulness-based therapy is not appropriate for acute psychiatric situations requiring urgent specialist care (Keng et al., 2011; Monteiro et al., 2015; Van Dam et al., 2018). Furthermore, the evidence base itself also has important limitations. Many studies are constrained by small sample sizes, short follow-up periods, heterogeneous intervention formats, variation in comparator conditions, and inconsistent outcome measures. In particular, different studies often assess distinct constructs, such as tinnitus-related distress, mood, stress, or quality of life, without clearly distinguishing these domains. This heterogeneity limits direct comparison across studies and makes it difficult to draw firm conclusions regarding overall efficacy, durability of benefit, or comparative effectiveness. Taken together, these methodological limitations warrant cautious interpretation of the current literature and highlight the need for more rigorous and standardized research in this field.

### 6.2 Future research directions

Future research should address the methodological and conceptual limitations of the current literature. Larger, better-controlled studies with clearer outcome classification are needed to determine more precisely the effects of mindfulness-based therapy on tinnitus-related distress, psychological functioning, quality of life, and tinnitus loudness. Studies should also examine which patient subgroups are most likely to benefit, given the heterogeneity of chronic tinnitus.

The potential value of combining mindfulness-based therapy with other approaches, including neuromodulatory interventions, warrants further investigation, but such work should be based on a clear mechanistic rationale and should assess whether combined treatment offers benefit beyond existing interventions. In parallel, future research should focus on improving adherence by addressing practical barriers such as time commitment, engagement difficulties, and drop-out. Digital or guided formats may be helpful, although these require further evaluation.

Greater conceptual clarity is also needed. Future studies should use consistent terminology, distinguish distress-related outcomes from tinnitus loudness, and avoid overstating preliminary mechanistic findings. This would improve both the interpretability of the evidence and the clinical application of mindfulness-based therapy in chronic tinnitus.

## 7 Conclusion

In conclusion, mindfulness-based therapy may represent a useful psychological intervention for some patients with chronic tinnitus, particularly in reducing tinnitus-related distress and improving emotional well being, coping, and quality of life. Current evidence does not support a consistent direct effect on tinnitus loudness, and this distinction should be maintained when interpreting reported outcomes. Although the available literature suggests potential benefit, the evidence base remains limited by methodological heterogeneity, variation in outcome constructs, and insufficient long-term high-quality data. Further well-designed studies are therefore needed to clarify the magnitude, durability, and clinical relevance of these effects in chronic tinnitus care.
